# Retrospective analysis of endocarditis patients to investigate the eligibility for oral antibiotic treatment in routine daily practice

**DOI:** 10.1007/s12471-020-01490-2

**Published:** 2020-09-17

**Authors:** J. C. Vroon, O. C. D. Liesdek, C. H. E. Boel, J. E. Arends, F. A. Niessen, H. C. van Heusden, M. J. Cramer, T. I. G. van der Spoel, S. A. J. Chamuleau

**Affiliations:** 1 Department of Cardiology, University Medical Center Utrecht, Utrecht, The Netherlands; 2 Department of Cardiothoracic Surgery, University Medical Center Utrecht, Utrecht, The Netherlands; 3 Department of Microbiology, University Medical Center Utrecht, Utrecht, The Netherlands; 4 Department of Internal Medicine and Infection Diseases, University Medical Center Utrecht, Utrecht, The Netherlands; 5 Department of Cardiology, Haga Hospital, The Hague, The Netherlands; 6 Department of Cardiology, Amsterdam University Medical Center, AMC/University of Amsterdam, Amsterdam, The Netherlands

**Keywords:** Endocarditis, Cardiac surgery, Oral treatment, Antibiotic treatment

## Abstract

**Background:**

According to the current guidelines of the European Society of Cardiology, patients with left-sided infective endocarditis are treated with intravenous antibiotics for 4–6 weeks, leading to extensive hospital stay and high costs. Recently, the Partial Oral Treatment of Endocarditis (POET) trial suggested that partial oral treatment is effective and safe in selected patients. Here, we investigated if such patients are seen in our daily clinical practice.

**Methods:**

We enrolled 119 adult patients diagnosed with left-sided infective endocarditis in a retrospective, observational study. We identified those that would be eligible for switching to partial oral antibiotic treatment as defined in the POET trial (e.g. stable clinical condition without signs of infection). Secondary objectives were to provide insight into the time until each patient was eligible for partial oral treatment, and to determine parameters of longer hospital stay and/or need for extended intravenous antibiotic treatment.

**Results:**

Applying the POET selection criteria, the condition of 38 patients (32%) was stable enough to switch them to partial oral treatment, of which 18 (47.3%), 8 (21.1%), 9 (23.7%) and 3 patients (7.9%) were eligible for switching after 10, 14, 21 days or 28 days of intravenous treatment, respectively.

**Conclusion:**

One-third of patients who presented with left-sided endocarditis in routine clinical practice were possible candidates for switching to partial oral treatment. This could have major implications for both the patient’s quality of life and healthcare costs. These results offer an interesting perspective for implementation of such a strategy, which should be accompanied by a prospective cost-effectiveness analysis.

**Electronic supplementary material:**

The online version of this article (10.1007/s12471-020-01490-2) contains supplementary material, which is available to authorized users.

## What’s new?

- Partial oral treatment is associated with favourable outcomes, low hospital mortality, a low reinfection rate and cost savings.
- One-third of 119 patients with left-sided infective endocarditis were in a clinically stable condition and eligible for switching to partial oral treatment, according to the criteria of the previously published Partial Oral Treatment of Endocarditis trial.
- Our study offers a perspective for implementation of this strategy, accompanied by a prospective cost-effectiveness analysis.

## Introduction

Currently, all patients with left-sided infective endocarditis are treated with intravenous antibiotics for 4–6 weeks, according to the guidelines of the European Society of Cardiology [1–3]. Adequate management of this fatal disease, with an in-hospital mortality rate of 15–40% [2–7], comprises early diagnosis with early initiation of intravenous bacteria-specific antibiotic treatment and surgical intervention if needed. The paradigm that patients with infective endocarditis must be treated with intravenous antibiotics under continuous clinical surveillance is changing due to new emerging evidence [1, 4], with a potential for partial outpatient treatment—intravenously or even orally.

Typically, intravenous treatment for 4–6 weeks requires prolonged hospital stay or a home-care management system. In additional, this treatment is associated with a reduced patient’s quality of life, higher risk of hospital-acquired complications resulting in deterioration of the patient’s clinical status, and increased healthcare costs [4, 5, 8, 9] Due to these disadvantages, interest has been raised in converting in-hospital intravenous antibiotic treatment to outpatient parenteral treatment in clinically stable patients 2 weeks after initiation of the intravenous treatment. Several studies have shown outpatient parenteral treatment is an effective and, importantly, safe option [10–14]. Furthermore, outpatient parenteral treatment reduces the risk of hospital-acquired complications, the psychological burden and the recovery time, and thus in-hospital costs [13–17]. Still, outpatient treatment requires practical home care and close monitoring of the therapeutic effects and side effects of the therapy. These logistic issues require dedicated care by home-visit nursing staff [1, 3, 4].

The drawbacks of outpatient antibiotic treatment for endocarditis can potentially be overcome by the introduction of a convenient alternative: partial oral treatment. Recently, Iversen et al. conducted a randomised controlled trial, the Partial Oral Treatment of Endocarditis (POET) trial, and reported that partial oral antibiotic treatment is non-inferior to intravenous therapy in patients in stable clinical condition [4].

Based on these promising results, we retrospectively evaluated patients diagnosed with left-sided infective endocarditis who presented to the University Medical Center Utrecht from 1 January 2016 until 1 December 2018. The main objective was to determine the number of patients in daily clinical practice that fulfil the POET criteria for clinically stable condition, making them eligible for safe partial oral antibiotic treatment.

## Methods

### Study design and population

In this retrospective, observational study, we reviewed the patient files of 119 consecutive adult patients with infective endocarditis that were discussed in the multidisciplinary endocarditis team; they received inpatient treatment at the University Medical Center Utrecht, the Netherlands and fulfilled the Duke criteria (definite or possible endocarditis) from 1 January 2016 until 1 December 2018.

**Fig. 1:**
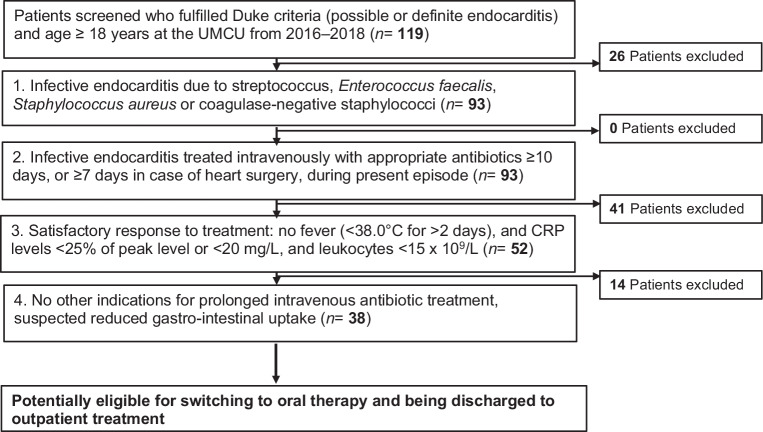
Flowchart displaying four criteria to identify patients in clinically stable condition to qualify for switching to partial oral treatment. A total of 38 patients fulfilled all criteria, while14 patients failed to meet the fourth criterion due to other indications for intravenous antibiotics. (*UMCU* University Medical Center Utrecht)

To evaluate which patients would have been, hypothetically, in stable clinical condition to qualify for oral treatment, we assessed them using the selection criteria used in the earlier POET trial of Iversen et al.: (1) positive blood cultures for the most commonly cultured microorganisms (i.e. streptococcus, *Enterococcus faecalis, Staphylococcus aureus* and coagulase-negative staphylococci); (2) patients had to be treated with intravenous antibiotics for at least 10 days or for at least 7 days after surgery; (3) evident clinical and biochemical response to the antibiotic therapy, i.e. no fever for at least 2 days consecutively, and a decrease in C‑reactive protein (CRP) levels below 25% of the peak CRP level at admission or CRP <20 mg/L and leukocytes <15 × 10^9^/L; and (4) absence of other indications for intravenous antibiotic treatment, such as pneumonia, or prolonged hospital stay due to complications. A flowchart is depicted in Fig. 1.

### Data collection and outcomes

We obtained all data and patient characteristics from the patient files. The primary outcome was the number of clinically stable patients that would have been eligible for switching to oral antibiotic treatment according to the criteria used in the recent POET trial. Secondary outcomes were: (1) evaluation of the individual time until a patient was eligible for partial oral treatment according to clinical and biochemical laboratory results; and (2) determination of other indications for longer hospital stay or intravenous antibiotic treatment due to complications.

## Results

A total of 119 adult patients with left-sided infective endocarditis were included, of whom 79% fulfilled the modified Duke criteria for definite endocarditis and 21% the criteria for possible endocarditis. Demographics and clinical characteristics of all patients are shown in Tab. 1.

**Table 1:** Characteristics of patients with left-sided infective endocarditis

Variable	Patients (*n* = 119)
Age, years (mean ± SD)	64.8 ± 13.1
Female gender	31 (26.1)
Comorbidity	
– Diabetes	20 (16.8)
– COPD/asthma	9 (7.6)
– Renal failure	7 (5.9)
– Cancer	29 (24.4)
Infective endocarditis according to Duke criteria	
– Possible	25 (21.0)
– Definite	94 (79.0)
Duke criteria	
– Pathological criteria	51 (42.9)
– Major	
a. Positive blood culture	78 (65.5)
b. Positive imaging	102 (85.7)
– Minor	
a. Predisposition (HC or IV)	52 (43.7)
b. Fever	79 (66.4)
c. Vascular phenomena	22 (18.5)
d. Immunological phenomena	4 (3.4)
e. Microbiological evidence	31 (26.1)
Microorganisms	
– Streptococcus	43 (36.1)
– *Enterococcus faecalis*	17 (14.3)
– *Staphylococcus aureus*	25 (21.0)
– Coagulase-negative staphylococci	8 (6.7)
Laboratory results at admission (mean ± SD)	
– Hemoglobin, mmol/L	7.1 ± 1.3
– CRP, mg/L	122.7 ± 105
– Leukocytes, ×10^9^/L	15.5 ± 25.3
– Creatinine, µmol/L	116.0 ± 90.2
Pre-existing device	
– Prosthetic heart valve	48 (40.3)
– Pacemaker	13 (10.9)
– Other known valve disease	
a. Bicuspid valve	5 (4.2)
b. Moderate or severe regurgitation	50 (42.0)
Cardiac involvement (left)	
– Mitral valve	38 (31.9)
– Aortic valve	47 (39.5)
– Mitral and aortic valve	15 (12.6)
– Pacemaker endocarditis	5 (4.2)
– Vegetation size >9 mm	28 (23.5)
Surgery	71 (59.7)
– EuroSCORE II (mean ± SD)	12.0 ± 13.9
Pacemaker lead extraction	1 (0.8)
Mortality	31 (26.1)
– <30 days after diagnosis	14 (45.2)
– <1 year after diagnosis	17 (54.8)
Mortality after surgery	14 (19.7)
– <30 days after surgery	7 (50.0)
– <1 year after surgery	7 (50.0)

All data are *n* (%) unless stated otherwise

*COPD* chronic obstructive pulmonary disease, *HC* heart condition, *IV* intravenous drug use, *CRP* C-reactive protein

The majority (74%) was male and their mean age was 65 years. Streptococcus was the most frequently identified microorganism, causing endocarditis in 43 cases (36%), followed by *S. aureus* in 25 patients (21%); 26 patients were infected by another causative pathogen (*n* = 18) or had consistently negative blood cultures (*n* = 8). The mean (±standard deviation) CRP level at admission was 123 mg/L (±105) and the mean leukocyte count was 15.5 × 10^9^/L (±25.3). The aortic valve was affected in 47 patients and the mitral valve in 38 patients; in 15 patients, both the aortic and mitral valve were infected. In 14 patients, imaging was inconclusive, and they were diagnosed with endocarditis based on other Duke criteria.

Thirty of the 48 patients with a previously inserted prosthetic heart valve presented with prosthetic valve endocarditis. Five out of 13 patients with an implanted pacemaker were diagnosed with active pacemaker lead endocarditis; one of these 5 patients underwent a lead extraction. All 13 patients with or without pacemaker lead endocarditis were diagnosed by transthoracic echocardiography. Additional imaging consisted of transoesophageal echocardiography and/or positron emission tomography combined with computed tomography (PET-CT) in case of inconclusive transthoracic echocardiography. Approximately 60% (*n* = 71) of all included patients underwent cardiac surgery; their mean EuroSCORE II was 12%.

The overall mortality was 26.1% (*n* = 31), of which 45.2% (*n* = 14) died within 30 days after endocarditis was diagnosed and 54.8% (*n* = 17) within 1 year after diagnosis. A total of 14 patients (19.7%) died after surgery, of whom 7 died within 30 days after surgery and 7 within one year after surgery. According to the selection criteria, as depicted in Fig. 1, a group of 93 patients fulfilled the criterion of infection with an endocarditis-causing microorganism. All these patients were intravenously treated with antibiotics for at least 10 days during conservative treatment or for at least 7 days after surgery. Antimicrobial therapy was in all cases initiated on the same day endocarditis was diagnosed.

Of the 93 patients who fulfilled the first selection criterion, 52 (56%) had a sufficient clinical and biochemical response to therapy according to the third criterion. Eventually 38 patients (32% of 52) had no other indication for intravenous antibiotics or prolonged hospital stay and were clinically stable and ready to convert to oral treatment of the infective endocarditis (Fig. 1). After 10, 14 and 21 days of intravenous antibiotic treatment, 18 patients (47.3%), 8 patients (21.1%) and 12 patients (31.6%), respectively, were eligible for POET (Tab. 2; see also Electronic Supplementary Material). Of the 52 patients, 14 did not fulfil the final POET criterion, since they had complications requiring prolonged intravenous treatment and/or longer hospitalization (Tab. 3). Most common indications were acute renal failure and delirium.

**Table 2:** Patients with endocarditis eligible for partial oral antibiotic treatment

Criterion	Patients (*n* = 38)
No fever^a^ and CRP levels <25% of peak level^b^ (*n*)	19
No fever^a^ and CRP <20 mg/L and leukocytes <15 × 10^9^/L (*n*)	19
Days after starting intravenous treatment (*n* (%))	
– 10–13 days	18 (47.3)
– 14–20 days	8 (21.1)
– 21–27 days	9 (23.7)
– 28–33 days	3 (7.9)

*CRP* C-reactive protein

^a^Temperature <38 °C for at least 2 days

^b^Peak level is CRP level at day of admission

**Table 3:** Patients with endocarditis ineligible for partial oral antibiotic treatment^a^

Criterion	Patients (*n* = 14)
Endocarditis-related complications during treatment	
– Severe MI (reoperation)	1
– Infected knee prosthesis	1
Complications after surgery	
– Acute renal failure (CVVH)	4
– Epileptic insult	1
– Pneumonia/pneumothorax	4
– SAB/mycotic aneurysm	1
– Mediastinitis	1
Other indication for hospitalization	
– Continual bleeding/factor VIII deficiency	1
– Epidural abscess	1
– Unsafe swallowing function as result of CVA	1
– Delirium	4

*MI* myocardial infarction, *CVVH* continuous venovenous hemofiltration, *SAB* subarachnoid bleeding, *CVA* cerebrovascular accident

^a^Complications (during treatment or after surgery) and other indications for intravenous antibiotic treatment or longer hospitalization

## Discussion

In this retrospective study, we observed that a significant group of patients with left-sided endocarditis in daily clinical practice (32%) was eligible for partial oral treatment with antibiotics. We found a slightly higher percentage of patients qualifying as clinically stable than that in the POET trial (32% vs 20%). The median time to switching to oral treatment in the initial POET trial was 17 days (range: 12–24) [4], which compares to our findings (mean time: 14 days; range: 10–33), although the range may be a matter of debate. However, it should be noted that 41 patients of the group of 93 did not have a sufficient clinical and biochemical response to the therapy. They were not in a clinically stable condition because of persistently high infection parameters caused by for example *S. aureus* bacteraemia or other infections, or patients died shortly after the diagnosis of endocarditis because of endocarditis-related complications during therapy.

Guidelines recommend intravenous antimicrobial therapy for 4–6 weeks [2, 3, 18, 19]. Intravenous medication is considered to be more effective than oral treatment, because therapeutic concentrations are rapidly achieved in the blood. In addition, most complications arise in the first 2 weeks—the so-called initial or critical phase—which require adequate intravenous treatment [2–5, 20]. In our study, 21.8% of all endocarditis patients were in clinically stable condition and eligible for POET between 10 to 21 days after initiation of antibiotic therapy. However, a larger time frame should be considered. Consequently, by extending the time frame to 28 days, we identified an even larger group of clinically stable patients that may be eligible for switching to oral antibiotics (29.4%), which is clinically highly relevant.

Other recommendations for oral treatment have been addressed by several studies. Earlier research confirmed that the outcomes of partial oral treatment are non-inferior to the outcomes of intravenous treatment [4]. Observational studies examined the efficacy of several oral antibiotics in the treatment of endocarditis. It has been suggested that switching to oral treatment is an effective and safe alternative [1, 17, 19, 21, 22]. In addition, it has been reported that switching to oral antibiotics is a feasible treatment, associated with favourable outcomes, low hospital mortality, a low reinfection rate, and cost savings. More prospective studies are crucial to evaluate the alternative oral therapy in practice and prove its cost-effectiveness.

### POET in future research

There are a few aspects of POET to be considered before conducting such studies, especially regarding the exclusion criteria, in an effort to include more patients. First, only patients with left-sided endocarditis were included; however, other studies also examined switching to oral treatment in patients with right-sided endocarditis. These studies reported efficient and safe treatment with oral ciprofloxacin and rifampicin without a higher risk of reinfection and with less drug toxicity [17, 19, 23, 24]. Although infective endocarditis most frequently affects the left side of the heart, right-sided endocarditis is seen on a regular basis. In our study, 5 patients were excluded due to the presence of right-sided infective endocarditis, while 3 of them were hypothetically ready for oral treatment. Also, for patients with right-sided endocarditis, partial oral treatment is highly relevant, and they should be included in future studies.

Second, 4 out of 8 infective endocarditis patients with persistently negative blood cultures in our study were in a clinically stable condition and eligible for POET within 16 days after initiation of intravenous antibiotic treatment. No previous study has investigated oral antibiotic treatment of infective endocarditis patients with persistently negative blood cultures. Moreover, even now, the best choice of specific intravenous antibiotic treatment is not clear, due to scarce data in this vulnerable patient population. Despite the good clinical condition of these patients, proposing switching to oral treatment would result in too much uncertainty during treatment.

Finally, it is important to note that the antibiotic regimens in the POET trial are not commonly used in the Netherlands. Although it is necessary to adhere to the adage of using two antibiotics with high biological availability, other antibiotics will be chosen for the conversion to oral treatment, in consultation with the guidelines of the Dutch Working Party on Antibiotic Policy (*Stichting Werkgroep Antibiotica Beleid*).

### Study limitations

Our study has some limitations, especially related to the design of an observational, retrospective study. After trying to obtain all data, those of 9 patients were still missing. The missing data concerned CRP levels, leukocyte counts and body temperature. Due to the referral of patients to our hospital and the discharge to another hospital, no continuous data were directly available. These 9 patients were lost to follow up after the third criterion of the flowchart.

## Conclusion

This retrospective, observational study showed that one-third of patients who presented with left-sided endocarditis in daily clinical practice were possible candidates for switching to partial oral treatment. Obviously, this could have major implications for both the patient’s quality of life and healthcare costs. These results offer an interesting perspective for implementation of such a strategy, which should be accompanied by a proper prospective cost-effectiveness analysis.

## Caption Electronic Supplementary Material

Patients ready for POET. The cumulative number of patients (%) ready for POET as the days progress between 10 days and 33 days after starting treatment.
