# Supplementary material for: Retrospective analysis of endocarditis patients to investigate the eligibility for oral antibiotic treatment in routine daily practice
Source: Neth Heart J. 2020 Sep 17;29(2):105–10. doi: 10.1007/s12471-020-01490-2 (PMC7843713; doi:10.1007/s12471-020-01490-2)
Supplement: Supplementary file 1 — Patients ready for POET. The cumulative number of patients (%) ready for POET as the days progress between 10 days and 33 days after starting treatment. [file 12471_2020_1490_MOESM1_ESM.docx]

# Electronic Supplementary Material
